# The Impact of Operator’s Learning Curve on the Outcomes of an Off-the-Shelf Multi-Branched Endograft for Complex and Thoracoabdominal Aneurysms Repair

**DOI:** 10.3390/jcm15103686

**Published:** 2026-05-11

**Authors:** Enrico Gallitto, Antonio Cappiello, Gianluca Faggioli, Andrea Vacirca, Paolo Spath, Stefania Caputo, Chiara Mascoli, Carmine Poliseno, Marco Mattiacci, Pietro Fiorio, Rodolfo Pini, Mauro Gargiulo

**Affiliations:** 1 Vascular Surgery, Department of Medical and Surgical Sciences (DIMEC), University of Bologna, 40138 Bologna, Italy; enrico.gallitto@gmail.com (E.G.); gianluca.faggioli@unibo.it (G.F.); andrea.vacirca3@unibo.it (A.V.); carmine.poliseno@studio.unibo.it (C.P.); marco.mattiacci@studio.unibo.it (M.M.); pietro.fiorio@studio.unibo.it (P.F.); rudypini@gmail.com (R.P.); mauro.gargiulo2@unibo.it (M.G.); 2 Vascular Surgery Unit, IRCCS Sant’Orsola, Azienda Ospedaliero-Universitaria, 40138 Bologna, Italy; paolo.spath@gmail.com (P.S.); caputostefania@hotmail.it (S.C.); chiara.ma@yahoo.it (C.M.)

**Keywords:** thoracoabdominal aortic aneurysms, complex aortic aneurysms, learning curve, T-Branch, spinal cord ischemia, BEVAR, endovascular aortic repair

## Abstract

**Objectives**: The study objectives are to evaluate the impact of the operator’s learning curve on the outcomes of off-the-shelf branched endovascular aortic repair (OTS-BEVAR) in complex and thoracoabdominal (C/TAAAs) aneurysms. **Methods**: Patients who underwent OTS-BEVAR (T-Branch, Cook-Medical) between 2013 and 2023 were considered. The first and last 25 cases with at least 1 year of follow-up were clustered in two groups (early experience: E.Exp.; late experience: L.Exp.) and compared. Technical success (TS), intraoperative target visceral vessel (TVV) loss, spinal cord ischemia (SCI), 30-day mortality, and TVV instability at 1 year were assessed as primary outcomes. Procedure/fluoroscopy time, iodinated contrast media (ICM) amount and length of hospitalization were assessed as secondary outcomes. A *p* value < 0.05 was considered statistically significant. **Results**: Overall, 106 cases were analyzed. Technical success was higher in L.Exp. than E.Exp. (L.Exp.: 25/25 vs. E.Exp.: 17/25; *p*: 0.004). Moreover, intraoperative TVVs loss was higher in E.Exp. than L.Exp. (E.Exp.: 12/25 vs. L.Exp.: 0/25; *p*: 0.0001). There were no differences in terms of 30-day/in-hospital mortality (E.Exp.:4/25 vs. L.Exp.:4/25; *p*: 1), SCI (E.Exp.:2/25 vs. L.Exp.:1/25; *p*: 1), and paraplegia (E.Exp.: 2/25 vs. L.Exp.: 0/25; *p*: 0.48) between E.Exp. and L.Exp. Cases of L.Exp. had lower fluoroscopy [E.Exp.: 160(21) vs. L.Exp.:72(18) min; *p*: 0.003] and overall procedure time [E.Exp.: 518(156) vs. L.Exp.:400(130) min; *p*: 0.04], ICM [EE: 220(45) vs. L.Exp.: 138(61) mL; *p*: 0.005] and hospitalization [E.Exp.: 17(9) vs. L.Exp.: 12(4) days; *p*: 0.03] than cases of E.Exp. Finally, patients managed in L.Exp. had lower TVVs instability at 12 months than patients managed in the E.Exp. (E.Exp.: 8/25 vs. L.Exp.: 1/25; *p*: 0.04). **Conclusions**: Cases managed in L.Exp. have higher TS, lower intraoperative TVVs loss and instability at 1-year than cases of E.Exp. Moreover, procedural/fluoroscopy time, ICM, radiation exposure and hospitalization are lower in L.Exp. than E.Exp.

## 1. Introduction

Thoracoabdominal aortic aneurysms (TAAAs) are aneurysms involving both thoracic and abdominal segments of the aorta and are classified into different types according to their extent [1,2,3]. The incidence of this disease is difficult to determine because of its often-silent clinical course and limited epidemiological data available in the literature. According to Stoecker et al., TAAAs account for approximately 5 to 10% of all thoracic aortic aneurysms [4]. Their etiology is heterogeneous and includes genetic disorders, atherosclerotic degeneration, and post-dissection evolution. According to Laplace’s law, as the aneurysm enlarges, the aortic wall becomes thinner and wall stress increases, thereby increasing the risk of rupture, dissection, and mortality [3]. Historically, open surgical repair has been the mainstay of the treatment of these aneurysms. With the development of endovascular techniques, the management of these aneurysms has evolved, with an increase in the use of endovascular therapy with favorable outcomes compared with open repair [5].

Branched endovascular aneurysm repair (BEVAR) is nowadays a well-established strategy for the treatment of patients with thoracoabdominal aortic aneurysms (TAAAs) in the presence of anatomical aortic–iliac and target visceral vessels (TVVs) feasibility [3,6]. Both custom-made and off-the-shelf (OTS) devices are currently commercially available and may be used in elective as well as urgent settings [7,8,9].

In 2012, the Cook Zenith T-Branch endograft (Cook Medical, Bloomington, IN, USA) became the first OTS multibranched thoracoabdominal device commercially available in Europe, with proven and reproducible early- and mid-term outcomes [8,10].

Owing to the complexity of anatomical and clinical scenarios, these procedures are technically demanding, time-consuming, and associated with high procedural, perioperative and follow-up costs. The operator learning curve may play a key role in optimizing outcomes, particularly in such technically and clinically challenging interventions. Although hospital and surgeon case volume are well-known predictors of outcomes in aortic surgery [11], limited data are available in the literature regarding the use of thoracoabdominal multibranched OTS endograft and the impact of the operator learning curve.

The aim of this study was to report the outcomes of endovascular repair of TAAAs using an OTS multibranched endograft and to analyze the impact of the operators’ learning curve.

## 2. Materials and Methods

### 2.1. Study Design and Patient Selection

This was an observational, single-center, retrospective study, conducted without financial support from companies or other institutions.

All consecutive patients undergoing elective or urgent TAAAs repair using an OTS BEVAR (Cook Zenith T-Branch, Cook Medical, Bloomington, IN, USA) between 1 January 2013 and 30 June 2023 were prospectively collected, de-identified using a unique coding number, clustered into a dedicated electronic database and further retrospectively analyzed. All patients provided dedicated informed consent for the endovascular aortic procedure and their anonymous data analysis.

Two cohorts were defined: early experience (E.Exp) and late experience (L.Exp.) The E.Exp cohort included the first 25 patients treated in our series, whereas the L.Exp cohort included the last 25 procedures with available 1-year radiological follow-up.

### 2.2. Endograft Characteristics

The Cook Zenith T-Branch was the first commercially available OTS branched endovascular device for thoracoabdominal aortic aneurysm repair in Europe [12,13]. Detailed device characteristics and manufacturers’ instructions for use have been extensively described in previously published reports [10,14].

Briefly, the endograft is designed with four downward-facing branches for TVVs [celiac trunk (CT); superior mesenteric artery (SMA); renal arteries (Ras)] and is delivered through a 22-Fr delivery system. The endograft proximal diameter is 34 mm tapering to 18 mm distally, with a total body length of 202 mm [8,10,14].

The T-Branch is intended for use in a modular configuration in combination with a wide range of standard endografts, including tapered thoracic components and abdominal bifurcated devices [14,15]. The anatomical suitability of the T-Branch for endovascular repair of TAAAs ranges from 40% to 80% of cases otherwise managed with custom-made devices [12,13,16].

### 2.3. Preoperative Workup and Inclusion Criteria

Endograft sizing and procedural planning were performed using thoracoabdominal computed tomography angiography (CTA) and post-processing reconstructions on dedicated software (3Mensio—Vascular Imaging, Bilthoven, The Netherlands) by the same vascular surgeons who performed the procedures. The patency of the left subclavian and hypogastric arteries was preserved whenever feasible. The study included all patients referred to our center with an asymptomatic TAAAs, in cases in which the OTS solution did not require longer proximal aortic coverage than a custom-made endograft. We also included all patients referred to our center for urgent TAAAs or c-AAAs (including symptomatic aneurysms, contained rupture, peripheral embolization, or asymptomatic TAAAs with a maximum diameter ≥ 80 mm). Patients with free rupture were excluded, as were those in whom adequate proximal and distal sealing zones (cylindrical shape, length > 20 mm) could not be ensured, and those in whom the OTS solution was not anatomically feasible.

Balloon-expandable or self-expandable bridging stent-grafts were used for TVVs according to anatomical characteristics and physician preference. In cases with fewer than four TVVs, the exceeding branch was occluded using an Amplatzer vascular plug. Procedural details, as well as perioperative and postoperative management protocols, were accurately described in previous reports [8,14]. Since January 2016, all procedures have been performed in a hybrid operating room (Philips Allura, Groningen, The Netherlands).

### 2.4. Endpoints and Definitions

Early primary outcomes included technical success (TS), intraoperative TVVs loss, spinal cord ischemia (SCI) and 30-day/in-hospital mortality. Secondary early outcomes included procedure/fluoroscopy times, dose area product (DAP), volume of iodinated contrast media, and length of hospital stay. TVV’s instability at 1 year of follow-up was assessed as a late outcome.

Preoperative comorbidities, aneurysm anatomical classification, operative risk factors, and postoperative complications were recorded and classified according to the current Society for Vascular Surgery (SVS) reporting standards for endovascular repair of aneurysms involving the renal–mesenteric arteries [6].

### 2.5. Follow-Up

Laboratory assessment of renal, hepatic, and pancreatic function, along with thoracoabdominal CTA, was performed before discharge or within 30 days postoperatively. The post-discharge surveillance protocol included Doppler ultrasound (DUS) or contrast-enhanced DUS (CEUS) and CTA at 6 and 12 months, and yearly thereafter. CTA was systematically performed in cases of diagnostic doubts or to plan a reintervention.

All patients received dual antiplatelet therapy for the first 6 postoperative months.

### 2.6. Statistical Analysis

Continuous data were reported as a median and interquartile range (IQR), whereas categorical variables were expressed as frequencies. Overall survival, freedom from reinterventions, and TVVs patency were estimated by Kaplan–Meier analysis.

Comparisons between early and late experience groups were evaluated by Fisher’s exact test or Student’s *t*-test and Mann–Whitney U test or analysis of variance (ANOVA) for categorical and continuous variables, respectively. A *p* value < 0.05 was considered statistically significant.

All statistical analyses were performed by SPSS software, version 28.0 (SPSS Inc., Chicago, IL, USA).

## 3. Results

### 3.1. Overall

Out of 378 F/B-EVAR procedures, 106 cases (28%) were managed by a multibranched OTS BEVAR device and were included in the present study. Elective and urgent procedures accounted for 33 (31%) and 73 (69%) cases, respectively.

**Figure 1:**
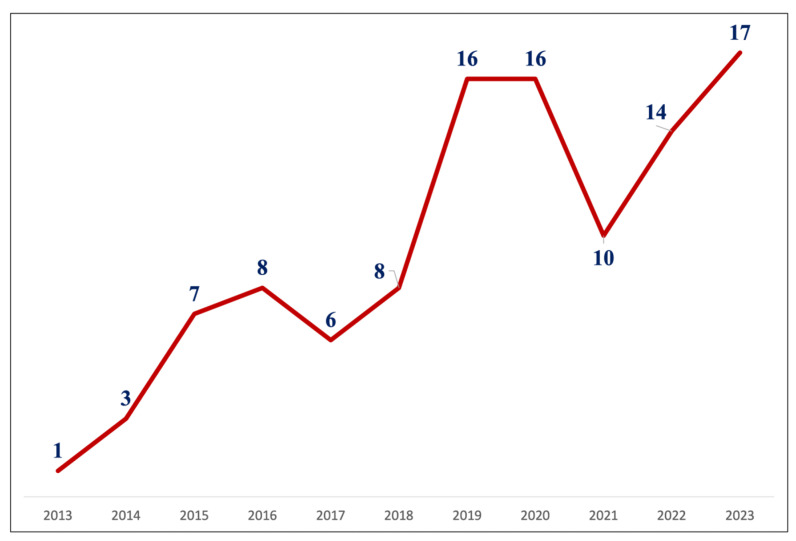
Distribution of T-branch cases per year.

Indications for urgent repair included ruptured, symptomatic, and large asymptomatic TAAAs in 28 (38%), 42 (88%), and 37 (50%) cases, respectively. Ten cases (9%) were classified as juxta-/pararenal abdominal aortic aneurysms (J/PAAAs), while 96 cases (91%) were TAAAs (Crawford extent I–III: 78–81%; IV: 18–19%). The annual distribution of procedures is shown in Figure 1.

Demographics, cardiovascular risk factors and preoperative comorbidities are reported in Table 1. A staged approach was adopted in 88 cases (83%) to mitigate the risk of SCI.

Overall technical success was achieved in 92 cases (elective: 88%, urgent: 86%). Reasons for technical failure are detailed in Supplementary Table S1.

Eleven (10%) patients suffered SCI (elective: 3%, urgent: 14%) with three (3%) cases of permanent paraplegia. Detailed information on SCI events, including timing, Tarlov grading, and anatomical and clinical characteristics, is provided in Supplementary Table S2. Five of the 11 patients (45%) who developed SCI died within 30 postoperative days.

Thirteen (12%) patients suffered respiratory complications (acute respiratory failure, pneumonia, and exacerbation of COPD), and 5 (5%) required temporary tracheostomy.

Overall, 30-day/in-hospital mortality was 18% (elective: 6%; urgent: 23%).

**Figure 2:**
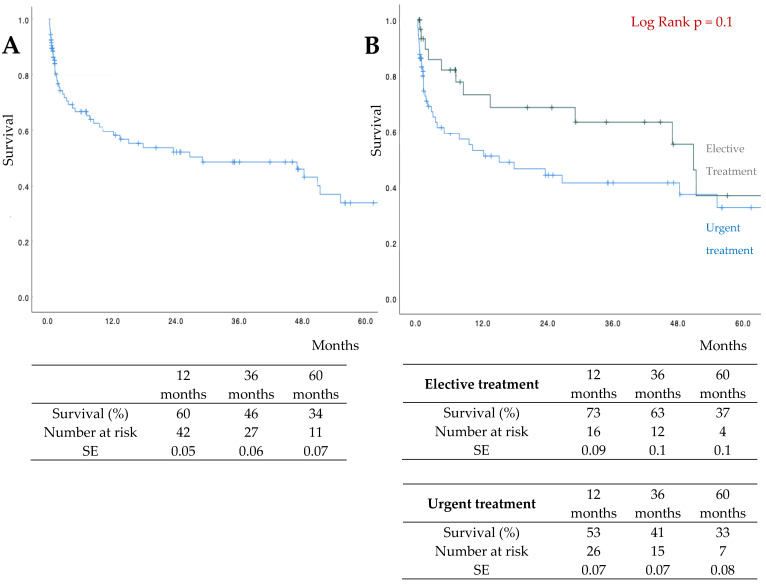
Freedom from (FF) survival by Kaplan–Meier analysis (**A**). Freedom from (FF) survival in elective group versus urgent group by Kaplan–Meier analysis (**B**).

The median follow-up was 24 months (IQR: 8–41). The estimated 3-year survival was 46% (elective: 63%; urgent: 41%) (Figure 2).

**Figure 3:**
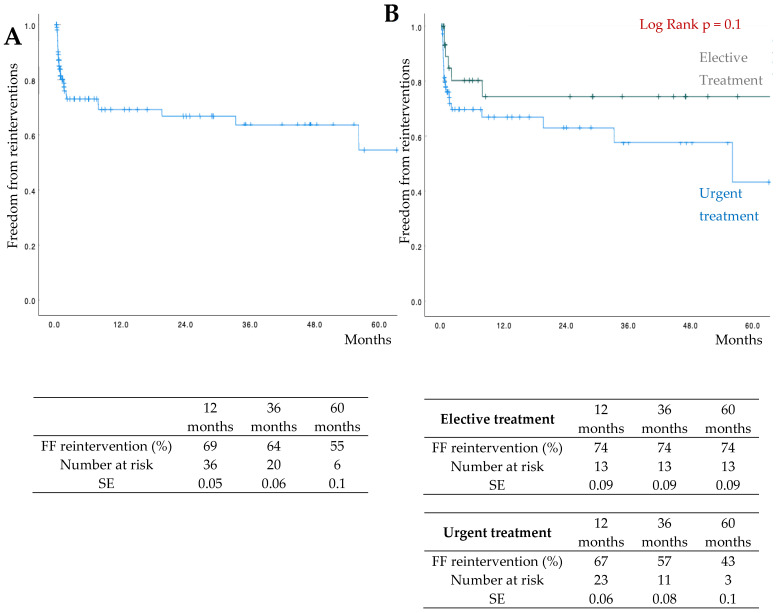
Freedom from (FF) reinterventions by Kaplan–Meier analysis (**A**). Freedom from (FF) reinterventions in elective group versus urgent group by Kaplan–Meier analysis (**B**).

The estimated 3-year freedom from reinterventions was 64% (elective: 74%; urgent: 57%) (Figure 3).

### 3.2. Early vs. Late Experience

No significant differences were observed between E.Exp and L.Exp groups in terms of demographics, cardiovascular risk factors, comorbidities, or preoperative clinical and anatomical characteristics (Table 2).

Technical success was significantly higher in the L.Exp group compared with the E.Exp group (L.Exp: 25/25 vs. E.Exp: 17/25; *p* = 0.004). Moreover, intraoperative TVVs loss was higher in the E.Exp group than in the L.Exp group (E.Exp: 12/25 vs. L.Exp: 0/25; *p* = 0.0001).

No significant differences were observed between the two groups in terms of 30-day/in-hospital mortality (E.Exp: 4/25 vs. L.Exp: 4/25; *p* = 1), SCI (E.Exp: 2/25 vs. L.Exp: 1/25; *p* = 1), or permanent paraplegia (E.Exp: 2/25 vs. L.Exp: 0/25; *p* = 0.48).

Procedures performed during the L.Exp were associated with significantly reduced fluoroscopy time (E.Exp: 160(21) vs. L.Exp:72(18) min; *p* = 0.003), overall procedure time (E.Exp: 518(156) vs. L.Exp:400(130) min; *p* = 0.04), DAP (E.Exp: 750887(1678411) vs. L.Exp: 608920(458994) mGy/cm2; *p* = 0.040), iodinated contrast volume (E.Exp: 220(45) vs. L.Exp: 138(61) mL; *p* = 0.005), and hospitalization (E.Exp: 17(9) vs. L.Exp: 12(4) days; *p* = 0.03) than cases of E.Exp.

Finally, patients managed in L.Exp demonstrated a significantly lower rate of TVV instability at 12 months compared with those treated during the E.Exp (E.Exp: 8/25 vs. L.Exp: 1/25; *p* = 0.04) (Table 3).

## 4. Discussion

In the present article, we report a retrospective single-center analysis of 106 consecutive patients managed by Cook Zenith T-branch for CAAAs and TAAAs, with the aim of evaluating the impact of the operator learning curve on technical and clinical outcomes.

Overall technical success was 87%, while 30-day/in-hospital mortality and permanent paraplegia rates were 18% (elective: 6% vs. urgent: 23%) and 3%, respectively. The median follow-up was 24 months with an estimated 3-year survival, freedom from reinterventions, and freedom from TVVs instability of 46%, 64% and 81%, respectively.

These findings should be interpreted considering the high-risk profile of the study population, as 40% of patients presented with rupture, 17% were symptomatic, and 40% were octogenarians. Within this context, the reported outcomes may be considered acceptable and clinically meaningful.

Importantly, our results are consistent with previously published series evaluating the use of the T-branch for urgent CAAAs and TAAAs, supporting the reproducibility of this approach in high-risk settings [8,9,17,18,19,20,21].

Regarding the operator learning curve, we compared outcomes between the first 25 cases and the last 25 cases of our experience. No differences were observed in 30-day mortality or SCI. However, cases performed during the later experience demonstrated higher technical success and lower intraoperative TVV loss compared with those performed during the early phase.

Moreover, procedural and fluoroscopy time, DAP, iodinated contrast media administration, and length of hospital stay were significantly lower in cases performed during late experience. Finally, TVVs instability was lower in the most recent cases.

Following initial pioneering experiences, F/B-EVAR has become a widely available technology for the treatment of CAAAs and TAAAs, with proven and reproducible results in anatomically selected high-risk patients [3,6]. However, these procedures remain costly, time-consuming, and technically demanding; therefore, they should be centralized in dedicated high-volume aortic centers [11,22,23].

The importance of the operators’/teams’ learning curve plays a key role in optimizing both technical and clinical outcomes, particularly considering the expected wider diffusion of F/B-EVAR procedures in the near future, driven by their satisfactory early- and mid-term outcomes. Previous dedicated papers have consistently demonstrated that increasing operator experience positively influences procedural efficiency as well as clinical outcomes [11].

In 2016, Starnes et al. reported the outcomes of 136 FEVAR procedures performed by a single surgeon between 2007 and 2015. Despite a progressive increase in case complexity over time, a significant reduction in perioperative mortality and major adverse events (MAEs) was observed, decreasing from 23% in the first quartile (Q1) to 9% in the fourth quartile (Q4). In parallel, procedural efficiency improved markedly, with procedural time decreasing from 223 min in Q1 to 150 min in Q4, and fluoroscopy time decreasing from 59 min to 32 min [24].

It is important to note that this series also included physician-modified endografts, which require an additional, specific learning curve related to on-table modification, further emphasizing the impact of operator experience on outcomes [24].

In 2020, Mirza et al. retrospectively reviewed 334 consecutive patients who underwent F-BEVAR between 2007 and 2016 for J/PAAAs and TAAAs. Despite an increasing proportion of TAAAs and TVVs incorporated into the graft over the study period, the authors demonstrated a significant reduction in 30-day mortality (6% in Q1 vs. 0% in Q4; *p* < 0.04) and in the rate of MAEs (60% in Q1 vs. 29% in Q4; *p* < 0.001).

Moreover, there was a significant decline in estimated blood loss (1358 + 1517 mL in Q1 vs. 486 + 520 mL in Q4; *p* < 0.001), total operative time (325 + 116 min in Q1 vs. 248 + 92 min in Q4; *p* < 0.001), total fluoroscopy time (121 + 59 min in Q1 to 85 + 39 min in Q4; *p* < 0.001), contrast volume (201 + 92 mL in Q1 to 160 + 61 mL in Q4; *p* = 0.002), and radiation dose (4141 + 2570 mGy in Q2 vs. 2543 + 1895 mGy in Q4; *p* < 0.001) [25].

Warmerdam et al. reported on 90 cases of complex endovascular aortic repair performed between 2013 and 2021. Patients were divided into three temporal groups, and the authors demonstrated a progressive reduction in overall procedural time, length of hospital stay, and cardiac complications over time. No significant differences in mortality or MAEs were observed; however, these findings should be interpreted considering the increasing procedural complexity during the study period [26].

In the present experience, we deliberately focused on an off-the-shelf BEVAR device, as its standard design minimizes potential selection bias related to device configuration and anatomical variability. This approach allows for a more homogeneous study population in terms of graft characteristics and anatomical applicability. Moreover, evaluating the impact of the learning curve with an off-the-shelf device is particularly relevant, given its rapid availability and suitability for prompt repair across different centers.

A similar study was reported by Squizzato et al., who analyzed the impact of the learning curve associated with the E-nside off-the-shelf device using data from the Italian multicenter registry. The authors demonstrated that, despite the increase in technical complexity in later cases, characterized by a higher proportion of post-dissection TAAAs, narrower lumens, and a greater use of a total transfemoral approach, there was a lower incidence of intraoperative adverse events compared with early experience (11% vs. 23%).

However, no significant differences were observed in procedural time, contrast media volume, or radiation exposure, nor were there differences in 30-day mortality, MAEs, or 2-year freedom from endograft instability, target artery instability, and target artery patency. The interpretation of these findings should take into account the intrinsic limitations of the study design, as the analysis included 225 cases from 26 centers, with a mean case volume of only 8.3 procedures per/center. This relatively low individual center volume may be insufficient to capture a true and measurable benefit related to the learning curve in terms of clinical and technical outcomes [27].

In our series, we reported a single-center, 10-year experience, with all cases planned by the same operator and performed by the same surgical team composed of two senior and two young consultants. According to our findings, we were able to demonstrate a significant reduction in key procedural metrics, including operative time, contrast media administration, radiation exposure, and length of hospital stay. Similar improvements were also observed in technical success, intraoperative target-artery loss, and 1-year target-artery instability.

It should be acknowledged that comparison between early and late experience over a 10-year period may also be influenced by the evolution of ancillary materials, such as newer-generation bridging stents, catheters, and adjunctive tools of a hybrid operating room. Nevertheless, it is noteworthy that major clinical outcomes, such as SCI and 30-day mortality, did not differ significantly between the early and late experience groups.

A comparable analysis was previously reported by Spanos et al. in a smaller cohort, in which outcomes in the late experience were also superior to those observed during the early phase [21].

Several limitations of the present study warrant consideration. This is a single-center, retrospective analysis with a relatively limited sample size and follow-up duration.

Due to the retrospective nature of the study, some patients’ characteristics (e.g., cardiovascular risk factors like physical activity, family heritage, and chronic inflammatory diseases) are missed. Moreover, while the center-level learning curve was evaluated, the individual operator learning curve was not specifically analyzed. Finally, procedural settings differed between the study periods; in fact, part of the early experience cases were performed using a mobile C-arm, whereas all later cases were conducted in a hybrid operating room, a factor that likely had a relevant impact on procedural efficiency and outcomes. It should also be considered a limitation of the study that materials, such as stent grafts, have evolved over the years and may have influenced the outcomes. It should also be noted that these are complex treatments requiring extensive use of devices, prolonged hospital stays, and high costs. These factors make the centralization of such patients in dedicated centers necessary. 

## 5. Conclusions

This single-center study demonstrates that Cook Zenith T-Branch is a feasible and effective solution for treating high-risk CAAAs and TAAAs, including urgent cases, achieving acceptable technical success (87%), 30-day mortality (18%), and permanent paraplegia rates (3%).

Despite increasing procedural complexity over the study period, the operator learning curve significantly enhanced procedural efficiency, reducing procedural/fluoroscopy times, radiation exposure, iodinated contrast media volume and hospitalization; as well as technical outcomes, including technical success and TVVs loss/instability rates. Conversely, no differences were observed in 30-day/in-hospital mortality, MAEs or SCI.

These findings underscore the importance of centralizing c-AAAs and TAAAs in dedicated high-volume centers to optimize both technical and clinical outcomes. However, larger series and further study with a formal learning curve model are necessary to validate this data.

## Figures and Tables

**Table 1:** Demographic, cardiovascular risk factors and preoperative comorbidities.

	N	%
Male	80	75
Hypertension	103	97
Active tobacco use	37	35
History of tobacco abuse	43	41
Dyslipidaemia	77	73
Diabetes	13	12
Chronic obstructive pulmonary disease	40	38
Coronary artery disease	36	34
Peripheral artery obstructive disease	17	16
Atrial fibrillation	12	11
Chronic kidney disease	52	49
History of stroke	13	12
Body Mass Index > 30 kg/m^2^	12	11
ASA ^1^ score 3	38	36
ASA ^1^ score 4	68	64
	**Mean**	**SD ^2^**
Age (years-old)	74	8
Aneurysm diameter (mm)	74	18

^1^ American Society of Anesthesiologists. ^2^ Standard deviation.

**Table 2:** Comparison of baseline demographics, cardiovascular risk factors, comorbidities, and anatomical features between early experience and late experience cohorts.

	Early Experience	Late Experience	*p*
	n—%	n—%	
Male	18–72	19–76	1
Hypertension	25–100	22–88	0.23
Active tobacco use	13–52	11–44	0.78
History of tobacco abuse	7–28	8–32	
Dyslipidaemia	18–72	19–76	1
Diabetes	3–12	3–12	1
Chronic obstructive pulmonary disease	15–60	10–40	0.26
Coronary artery disease	7–28	7–28	1
Peripheral artery obstructive disease	2–8	2–8	1
Atrial fibrillation	3–12	3–12	
Chronic kidney disease	12–48	10–40	0.78
History of stroke	3–12	3–12	1
Body Mass Index > 30 kg/m^2^	3–12	2–8	1
ASA ^1^ score 3	13–52	13–52	1
ASA ^1^ score 4	12–48	12–48	1
Post-dissection TAAAs	2–8	2–8	
Complex abdominal aortic aneurysm	2–8	5–20	0.41
Type IV TAAAs	5–20	7–28	0.74
Type I-II-III TAAAs	18–72	13–52	0.24
Mono-lateral Hypogastric occlusion	0–0	3–12	
Previous aortic surgery	13–52	13–52	1
Treatment in urgent setting	14–56	19–76	0.23
	**Mean–SD ^2^**	**Mean–SD ^2^**	***p***
Age (years old)	72 ± 11	76 ± 10	0.09
Aneurysm diameter (mm)	65 ± 21	66 ± 20	1

^1^ American Society of Anesthesiologists. ^2^ Standard deviation.

**Table 3:** Target arteries’ instability and reinterventions.

Patients	Group	Tas ^1^ Event	Time (months)	Reintervention	Results
1	E.Exp	RRA occlusion	1	Yes	Sealed
2	E.Exp	Endoleak IIIc from CT ^2^	63	Yes	Sealed
3	E.Exp	Endoleak Ic from CT ^2^	7	Yes	Sealed
4	E.Exp	Bleeding of LRA ^3^	1	Yes	Sealed
5	E.Exp	Endoleak Ic from LRA ^3^	1	Yes	Sealed
6	E.Exp	Occlusion of RRA ^4^	5	Yes	Sealed
7	E.Exp	Endoleak IIIc from SMA ^5^	96	Yes	Sealed
8	E.Exp	Endoleak IIIc from CT ^2^	20	Yes	Sealed
1	L.Exp	Occlusion of RRA ^4^	1	Yes	Not sealed

^1^ Target arteries. ^2^ Celiac trunk. ^3^ Left renal artery. ^4^ Right renal artery. ^5^ Superior mesenteric artery.

## Data Availability

The authors will provide the raw data that underpins the article upon request.

## Supplementary Materials

The following supporting information can be downloaded at: https://www.mdpi.com/article/10.3390/jcm15103686/s1, Table S1: Cause of technical failure; Table S2: Cases of SCI with timing, Tarlov’s grading, anatomical and clinical details.

## Abbreviations

The following abbreviations are used in this manuscript:

OTS	Off-the-shelf
BEVAR	Branched endovascular aortic repair
CAAA	Complex abdominal aortic aneurysm
TAAA	Thoracoabdominal aortic aneurysm
E.Exp.	Early experience
L.Exp.	Late experience
TS	Technical success
TVVs	Target visceral vessels
SCI	Spinal cord ischemia
ICM	Iodinated contrast media
CT	Celiac trunk
SMA	Superior mesenteric artery
Ras	Renal arteries
CTA	Computered tomography angiography
SVS	Society for Vascular Surgery
DUS	Doppler ultrasound
CEUS	Contrast-enhanced Doppler ultrasound
IQR	Interquartile range
MAEs	Major adverse events
ASA	American society of anesthesiologists
SD	Standard deviation
TAs	Target arteries
RRA	Right renal artery
LRA	Left renal artery
