# Supplementary material for: The Impact of Operator’s Learning Curve on the Outcomes of an Off-the-Shelf Multi-Branched Endograft for Complex and Thoracoabdominal Aneurysms Repair
Source: J Clin Med. 2026 May 11;15(10):3686. doi: 10.3390/jcm15103686 (PMC13207386; doi:10.3390/jcm15103686)
Supplement: Supplementary file 1 [file jcm-15-03686-s001.zip › jcm-4270853-supplementary.pdf]

Supplementary Materials:

Technical failure (14 - 100%)	**N**	**%**
Target visceral vessel loss	8	57
Necessity of iliac conduit	1	7
Inter-step mortality	4	29
Nephrectomy due to renal bleeding	1	7

Table S1. Cause of technical failure.

**Patients**	**Urgent**	**Type of aneurysm**	**Tarlov scale**	**Time (days)**	**CSF drain**	**Tarlov scale at discharge**
1	Yes	Type II TAAA	3	0	Prophylactic	0
2	Yes	Type I TAAA	0	0	No	0
3	Yes	Type II TAAA	4	1	No	4
4	Yes	Type II TAAA	4	0	Prophylactic	5
5	Yes	Type IV TAAA	4	5	No	5
6	Yes	Type II TAAA	4	0	Prophylactic	4
7	Yes	Type II TAAA	3	1	Prophylactic	4
8	Yes	Type II TAAA	1	0	Therapeutic	1
9	Yes	Type IV TAAA	3	1	Therapeutic	4
10	Yes	Type II TAAA	0	1	Prophylactic	0
11	No	Type III TAAA	4	8	Therapeutic	5

Table S2. Cases of SCI with timing, Tarlov's grading, anatomical and clinical details.
